# Supplementary material for: Analysis of follow-up data in large biobank cohorts: a review of methodology
Source: Front Genet. 2025 Jun 25;16:1534726. doi: 10.3389/fgene.2025.1534726 (PMC12238046; doi:10.3389/fgene.2025.1534726)
Supplement: Supplementary file 1 [file DataSheet1.pdf]

Supporting Information for Analysis of follow-up data in large biobank cohorts: a review of methodology by **Anastassia Kolde, Merli Koitmäe**, Meelis Käärik, Märt Möls and Krista Fischer

May 14, 2025

1 Modeling parental survival using offspring genotype

Joshi et al have assumed [22] that the expected allelic effect of one allele in offspring is 0.5 and the survival function is in the form of

$$S_P(t|X_o) = (S_{0P}(t))^{e^{0.5X_o\beta}},$$

where S_P is parental survival function, S_{0P} is the parental baseline survival, X_O denotes the offspring allele count and β is the effect size of the allele count.

Assume that the Cox proportional hazard (CPH) model holds. If parental genotype and survival times were known, then it would be true that

$$S_P(t|X_P) = (S_{0P}(t))^{e^{X_P\beta}},$$

where S denotes survival function and X_P is the matrix of parental allele counts. Let us denote the minor allele frequency with p and assume that it stays constant over time. For allele count X it holds that

$$P(X = 0) = (1 - p)^2$$
$$P(X = 1) = 2p(1 - p)$$
$$P(X = 2) = p^2.$$

Knowing parental genotypes, we can calculate probability of offspring allele counts X_o.

Parental AC	0	1	2
0	$P(X_o = 0) = 1$<br>$P(X_o = 1) = 0$<br>$P(X_o = 2) = 0$	$P(X_o = 0) = 0.5$<br>$P(X_o = 1) = 0.5$<br>$P(X_o = 2) = 0$	$P(X_o = 0) = 0$<br>$P(X_o = 1) = 1$<br>$P(X_o = 2) = 0$
1	$P(X_o = 0) = 0.5$<br>$P(X_o = 1) = 0.5$<br>$P(X_o = 2) = 0$	$P(X_o = 0) = 0.25$<br>$P(X_o = 1) = 0.5$<br>$P(X_o = 2) = 0.25$	$P(X_o = 0) = 0$<br>$P(X_o = 1) = 0.5$<br>$P(X_o = 2) = 0.5$
2	$P(X_o = 0) = 0$<br>$P(X_o = 1) = 1$<br>$P(X_o = 2) = 0$	$P(X_o = 0) = 0$<br>$P(X_o = 1) = 0.5$<br>$P(X_o = 2) = 0.5$	$P(X_o = 0) = 0$<br>$P(X_o = 1) = 0$<br>$P(X_o = 2) = 1$

Table 1: Offspring allele count probability in different parental scenarios.

Now using the Law of Total Probability we can derive conditional probabilities that offspring allele count X_o is l, given that one parental allele count X_p is k,

$$P(X_o = l|X_p = k) = \sum_{j=0}^{2} P(X_o = l|X_p = k, X_j = j)P(X_j = j),$$

where X_j denotes the allele count of the other parent. Now, we can see that

$$\begin{aligned}P(X_o = 0|X_p = 0) = \sum_{j=0}^{2} P(X_o = 0|X_p = 0, X_j = j)P(X_j = j) = \\ = P(X_o = 0|X_p = 0, X_j = 0)P(X_j = 0)+ \\ + P(X_o = 0|X_p = 0, X_j = 1)P(X_j = 1)+ \\ + P(X_o = 0|X_p = 0, X_j = 2)P(X_j = 2) = \\ = 1 \cdot (1-p)^2 + 0.5 \cdot 2p(1-p) + 0 \cdot^2 = \\ = (1-p)^2 + p(1-p) = 1 - p.\end{aligned}$$

Analogically we cand find all the other conditional probabilities, which results in the following table

Parent X_p \ Offspring X_o	0	1	2
0	1-p	p	0
1	0.5(1-p)	0.5	0.5p
2	0	1-p	p

Table 2: Offspring allele count probability, conditional on the parental allele count.

Thus using Bayes' theorem we can derive the equation for parental survival function, conditional on the offspring allele count

$$\begin{aligned}S_p(t|X_o) &= P(T_p > t|X_o = l) = \\ &= \sum_{k=0}^{2} P(T_P > t|X_o = l, X_p = k)P(X_p = k|X_o = l) = \\ &= \sum_{k=0}^{2} P(T_p > t|X_p = k)\frac{P(X_o = l|X_p = k)P(X_p = k)}{P(X_o = l)} = \\ &= \frac{1}{P(X_o = l)} \sum_{k=0}^{2} S_p(t|X_p)P(X_o = l|X_p = k)P(X_p = k) = \\ &= \frac{1}{P(X_o = l)} \sum_{k=0}^{2} (S_{0p}(t))^{e^{k\beta}} P(X_o = l|X_p = k)P(X_p = k).\end{aligned}$$

From this it is easy to see that

$$\begin{aligned}S_p(t|X_o = 0) &= \frac{1}{(1-p)^2}(S_{0p}(t)(1-p)^2(1-p)+ \\ &+ S_{0p}(t)^{e^{\beta}} 2p(1-p)0.5(1-p)) = \\ &= S_{0p}(t)(1-p) + S_{0p}(t)^{e^{\beta}} p \\ S_P(t|X_o = 1) &= \frac{1}{2p(1-p)}(S_{0p}(t)(1-p)^2 p + S_{0p}(t)^{e^{\beta}} 2p(1-p)0.5+ \\ &+ S_{0p}(t)^{e^{2\beta}} p^2(1-p)) = \\ &= \frac{1}{2}(S_{0p}(t)(1-p) + S_{0p}(t)^{e^{\beta}} + S_{0p}(t)^{e^{2\beta}} p) \\ S_p(t|X_o = 2) &= \frac{1}{p^2}(S_{0p}(t)^{e^{\beta}} 2p(1-p)0.5p + S_{0p}(t)^{e^{2\beta}} p^2 p) = \\ &= S_{0p}(t)^{e^{\beta}}(1-p) + S_{0p}(t)^{e^{2\beta}} p\end{aligned}$$

Bias if $X_o = 0$:

$$S_{0p}(t)(1-p) + S_{0p}(t)^{e^{\beta}} p - S_{0p}(t) = p(S_{0p}(t)^{e^{\beta}} - S_{0p}(t))$$

Bias if $X_o = 1$:

$$\frac{1}{2}(S_{0p}(t)(1-p) + S_{0p}(t)^{e^{\beta}} + S_{0p}(t)^{e^{2\beta}} p) - S_{0p}(t) e^{0.5\beta}$$

Bias if $X_o = 2$:

$$S_{0p}(t)^{e^{\beta}}(1-p) + S_{0p}(t)^{e^{2\beta}} p - S_{0p}(t)^{e^{\beta}} = p(S_{0p}(t)^{e^{2\beta}} - S_{0p}(t)^{e^{\beta}})$$

If $\beta \neq 0$ (and $p \neq 0$ and $S_{0p} \not\equiv 0$ and $S_{0p} \not\equiv 1$) the formula proposed by Joshi et al for parental survival function is biased.

2 Simulation study

2.1 Data-generation algorithm for unrelated individuals

The parameters for the simulated distribution of the time to event variable are chosen to match the corresponding estimates from the overall survival distribution in the Estonian Biobank data.
Step 1. We began by generating of two variables: the first, $X_1 \sim Bin(2, MAF)$, simulates the genotype variable, where MAF signifies the minor allele frequency of a SNP, while the second variable, denoted as $X_2 \sim N(0,1)$, represents a generic technical covariate.
Step 2. Survival time of the individuals was generated using Weibull distribution, $T \sim W(\lambda, \gamma)$. We chose the shape and scale parameters by the objective of generating survival times that closely approximate those observed in the Estonian Biobank. The survival time was set to be a linear combination of simulated technical covariate and genotype. The effect size β_1 for genotype was varied mainly in the domain of GWAS effect sizes, to range from 0 to 0.4 by 0.01. The effect size of the technical covariate was kept constant, $\beta_2 = 1$.
Step 3. We generated the year of birth for individuals in the population to be uniformly distributed between 1920 and 1990.
Step 4. For each individual we generated a personal recruitment year $Y_{\text{rec}} \sim \text{Unif}(2012, 2017)$. Individuals whose year of death, defined by $Y_{\text{death}} = Y_{\text{birth}} + T$ occurred before their recruitment year were excluded, thereby reproducing the real-world requirement that participants must be alive at enrollment and introducing left truncation (delayed entry). Administrative right censoring was imposed at a study cut-off year Y_{cut}, set to 2020, 2025, and 2070 to yield censoring rates of $\approx 90\%$, $\approx 80\%$, and $\approx 10\%$, respectively. The observed time is therefore $\tilde{T} = \min\big(Y_{\text{death}}, Y_{\text{cut}}\big) - Y_{\text{birth}}$ with event indicator $\delta = \mathbf{I}\{Y_{\text{death}} \leq Y_{\text{cut}}\}$.
The initial sample size was $N = 100\ 000$. For each simulation the final sample size was varying due to omission of random number of people in step 4. These steps were repeated 500 times.

2.2 Data-generation algorithm for related individuals

As for simulation of unrelated individuals, the parameters for the simulated distribution of the time to event variable are chosen to match the corresponding estimates from the overall survival distribution in the Estonian Biobank data. For related individuals, we used a family setting setup and included an additional covariate, a frailty term.
Step 1. We began by generating parental genotypes, $X_{ij} \sim Bin(1, MAF)$, where i represents a parent and $j = 1, 2$. Parental phenotypes from a normal distribution, $X_2 \sim N(24, 3)$. A shared family frailty term was also introduced, sampled from $X_3 \sim N(0,1)$. For each family, the number of offspring was determined using a Poisson distribution. Each child's genotype was a combination of randomly selected alleles from both parents, and their phenotype was a linear combination of the parental phenotypes. All siblings within a family shared the same family frailty term as a representation of unobserved shared risk factors.
Step 2. Survival time of the offsprings was generated using Weibull distribution, $T \sim W(\lambda, \gamma)$. We chose the shape and scale parameters by the objective of generating survival times that closely approximate those observed in the Estonian Biobank. The survival time was set to be a linear combination of simulated genotype, phenotype and a frailty termm. The effect size β_1 for genotype was varied mainly in the domain of GWAS effect sizes, to range from 0 to 0.4 by 0.05. The effect size of the phenotype and frailty was kept constant, $\beta_2 = 0.05$ and $\beta_3 = 0.5$ respectively.
Step 3. We generated the year of birth for offsprings in the population to be uniformly distributed between 1920 and 1990.
Step 4. For each offspring we generated a personal recruitment year $Y_{\text{rec}} \sim \text{Unif}(2012, 2017)$. Individuals whose simulated year of death $Y_{\text{death}} = Y_{\text{birth}} + T$ occurred before their recruitment year were excluded, thereby reproducing the real-world requirement that participants must be alive at enrollment and introducing left truncation (delayed entry). Administrative right censoring was imposed at a study cut-off year Y_{cut}, set to 2020, 2025, and 2070 to yield censoring rates of $\approx 90\%$, $\approx 80\%$, and $\approx 5\%$, respectively. The observed time is therefore $\tilde{T} = \min\big(Y_{\text{death}}, Y_{\text{cut}}\big) - Y_{\text{birth}}$ with event indicator $\delta = \mathbf{I}\{Y_{\text{death}} \leq Y_{\text{cut}}\}$.
The initial sample consisted of $N = 7\ 000$ families. For each simulation the final sample size was varying due to omission of random number of people in step 4. These steps were repeated 300 times.

2.3 Models for simulated data

2.3.1 The effect of timescale choice on bias and power

In Cox PH models we looked at three different timescale scenarios:

1. models with age since birth as timescale, ie only accounting for right-censoring and ignoring the left-truncation (denoted **timescale TB**), adjusted for X_2;

2. age- and X_2-adjusted models with time since recruitment as timescale (denoted **timescale TR+A**);

3. models with age as timescale, ie accounting for both right-censoring and left-truncation (denoted **timescale TA**), adjusted for X_2.

2.3.2 Utility of martingale residuals based approach

To compare the conventional Cox model and two-step MR approach, the following models were fit:

1. The full model

$$h(t;x) = h_0(t)e^{\beta_1 X_1 + \beta_2 X_2 + \ldots + \beta_n X_n}, \tag{1}$$

   where $h_0(t)$ is a baseline hazard function and X_1 represents genetic marker and $X_2 \ldots X_n$ all the other covariates using the conventional Cox partial likelihood approach for model-fitting.

2. Next we fit baseline CPH model where we account only for nongenetic covariates $h(t;x) = h_0(t)e^{\beta_2 X_2 + \ldots + \beta_n X_n}$, followed by calculation of the martingale residuals

$$\hat{M_i} = \delta_i - \hat{H_i}(y_i | X_2 = x_{i2}, \ldots, X_n = x_{in}),$$

   where δ_i the event indicator for the i-th subject (1 - died, 0 - survived at the end of follow-up) and $\hat{H_i}(y_i | X_2 = x_{i2}, \ldots, X_n = x_{in})$ is an estimate of the conditional expected value of δ_i for i-th subject for the total observation time y_i. As a final step in two-step approach, residuals, corrected for incidence, are regressed on the covariate of interest that was left out from the baseline CPH model

$$M^* = \alpha + \beta_1^* X_1, \tag{2}$$

   where $M^* = M \cdot N / \sum_i (I(\delta_i = 1))$, $I(\delta_i = 1) = [\delta_i = 1]$, and N is sample size, whereas β_1^* should approximate β_1 from (1).

The performance of a two-step MR approach was evaluated under different censoring rates (90%, 80%, and 10%) and MAFs (0.05, 0.4). The desired censoring rates were achieved by altering the length of the follow-up period. For investigating related subjects, a 5% censoring rate was used instead of 10% to ensure comparable follow-up periods.

3 Figures

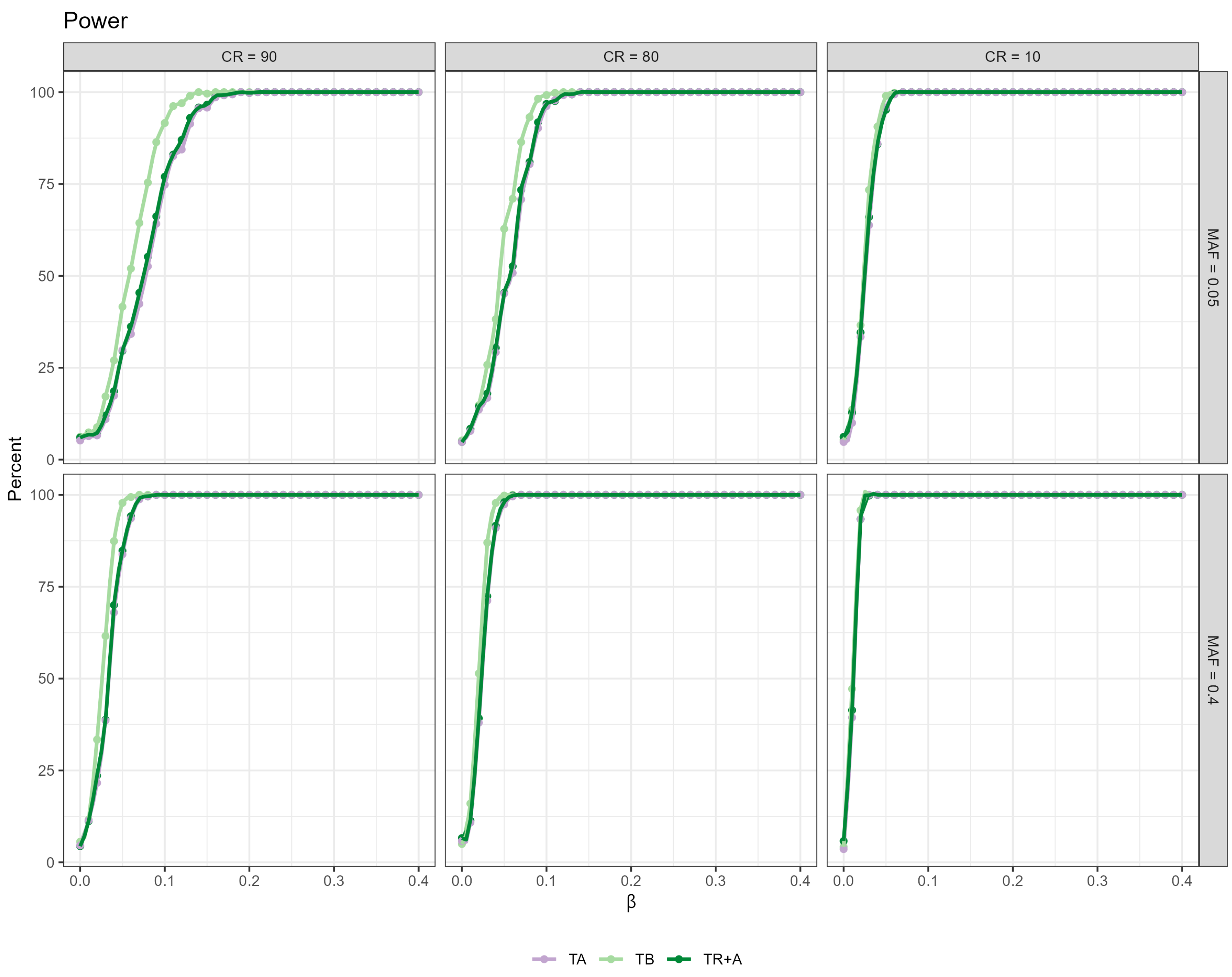

Figure 1: Power analysis under different censoring rates (CR) and minor allele frequencies (MAF) across three different timescale choices (TA, TB, TR+A).

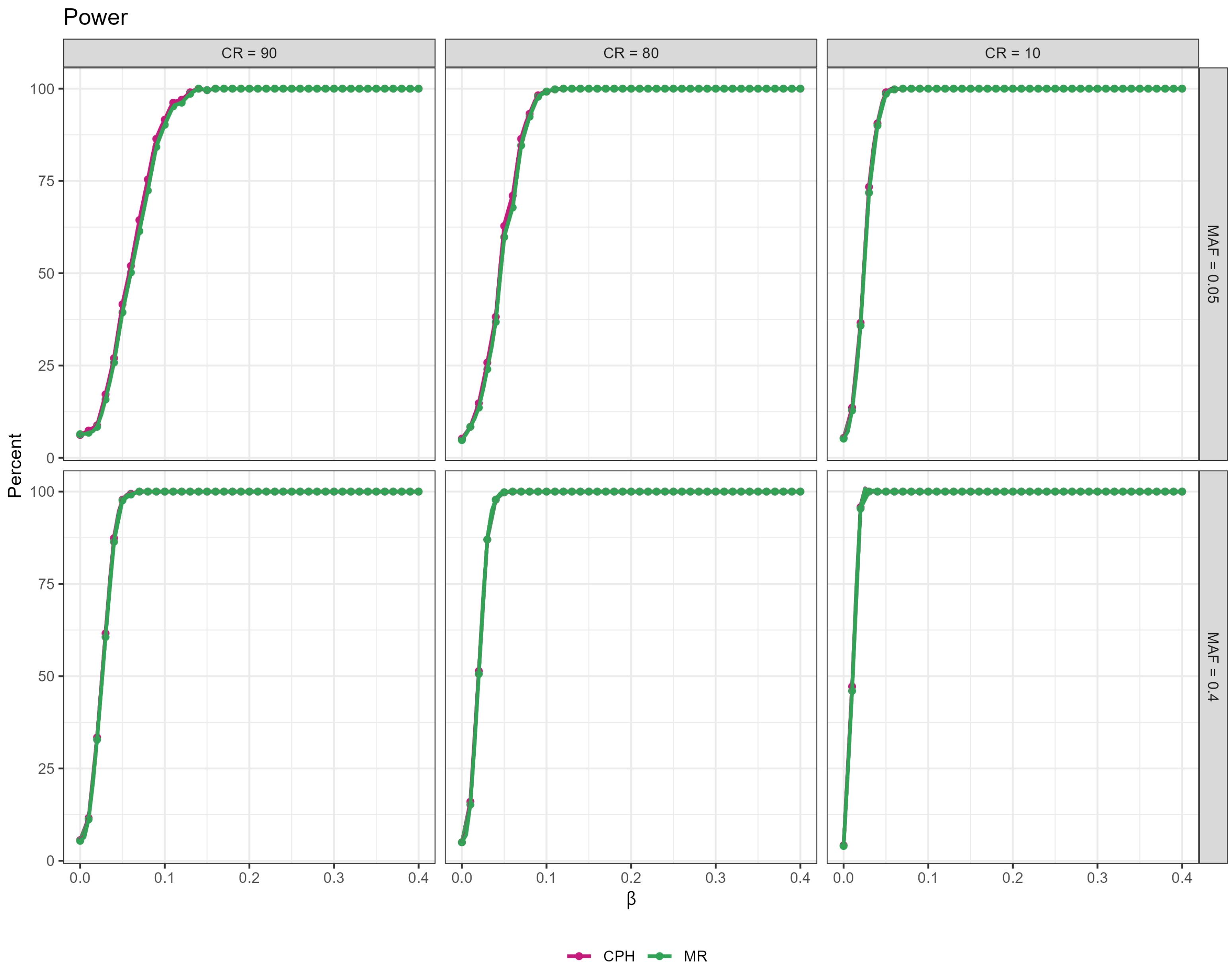

Figure 2: Power analysis under various censoring rates (CR) and minor allele frequencies (MAF), comparing β estimates using the CPH and MR approaches with age since birth as the timescale.

Table 3: Estimated Type I error rates across different timescales, censoring rates (CR), and minor allele frequencies (MAF). Each value represents the proportion of simulations (out of 500) in which the null hypothesis was incorrectly rejected at a significance level of $\alpha = 0.05$. The 95% confidence intervals were calculated using the Wilson method for binomial proportions.

**Timescale**	**CR**	**MAF**	**Type I Error Rate (95% CI)**
TB	CR = 90	MAF = 0.05	0.062 [0.044, 0.087]
TA	CR = 90	MAF = 0.05	0.052 [0.036, 0.075]
TR+A	CR = 90	MAF = 0.05	0.060 [0.042, 0.084]
TB	CR = 90	MAF = 0.4	0.056 [0.039, 0.080]
TA	CR = 90	MAF = 0.4	0.046 [0.031, 0.068]
TR+A	CR = 90	MAF = 0.4	0.044 [0.029, 0.066]
TB	CR = 80	MAF = 0.05	0.052 [0.036, 0.075]
TA	CR = 80	MAF = 0.05	0.048 [0.032, 0.070]
TR+A	CR = 80	MAF = 0.05	0.048 [0.032, 0.070]
TB	CR = 80	MAF = 0.4	0.050 [0.034, 0.073]
TA	CR = 80	MAF = 0.4	0.058 [0.041, 0.082]
TR+A	CR = 80	MAF = 0.4	0.066 [0.047, 0.091]
TB	CR = 10	MAF = 0.05	0.054 [0.037, 0.077]
TA	CR = 10	MAF = 0.05	0.048 [0.032, 0.070]
TR+A	CR = 10	MAF = 0.05	0.062 [0.044, 0.087]
TB	CR = 10	MAF = 0.4	0.042 [0.028, 0.063]
TA	CR = 10	MAF = 0.4	0.036 [0.023, 0.056]
TR+A	CR = 10	MAF = 0.4	0.058 [0.041, 0.082]

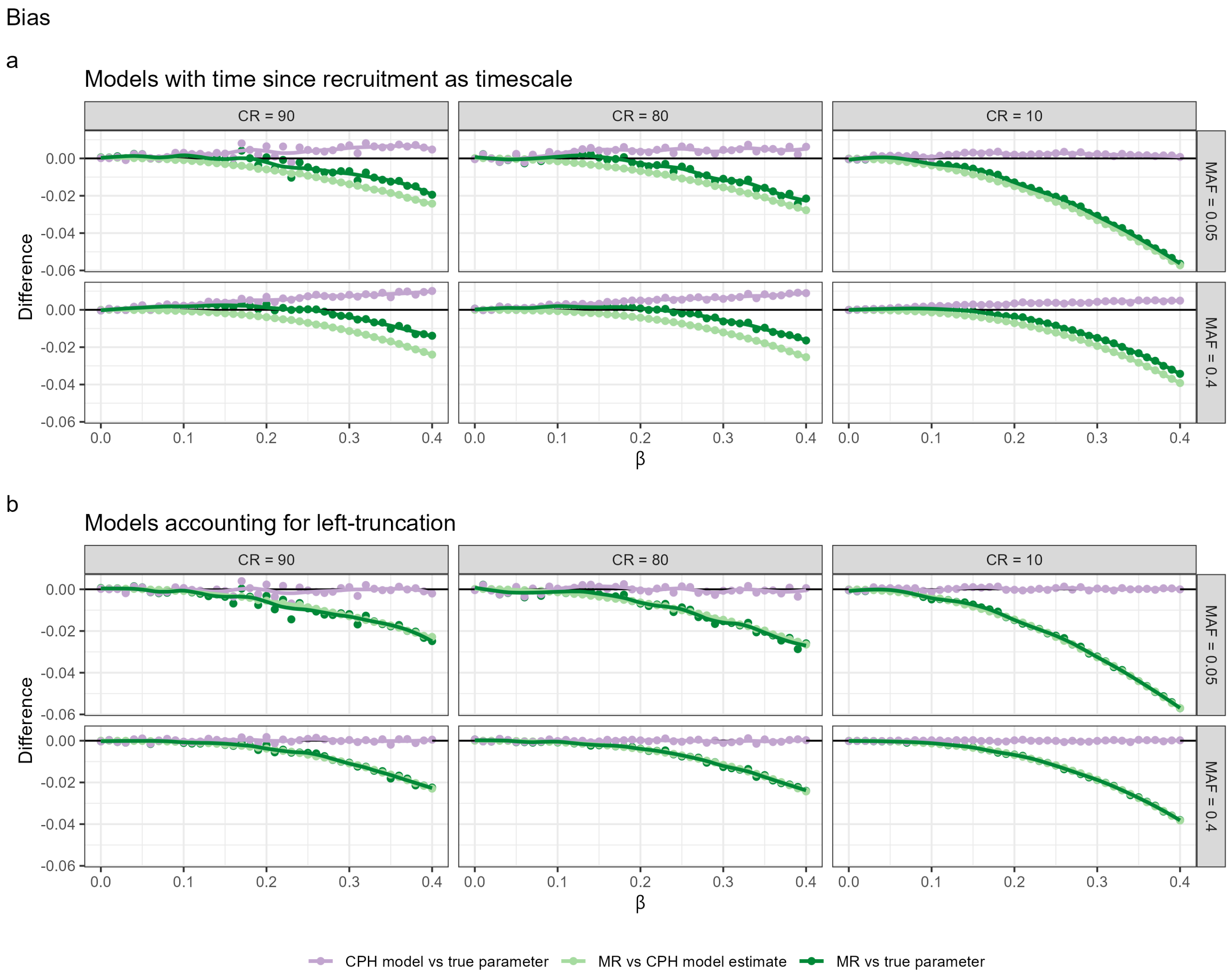

Figure 3: Bias for models using time since recruitment as the timescale (a) and models accounting for left-truncation (b), evaluated across different censoring rates and MAFs. Three different scenarios are compared: β estimate using MR approach vs true β *(MR vs β)*, β estimate using MR approach vs CPH estimate *(MR vs CPH model estimate)* and CPH model estimate vs true β *(CPH model vs β)*

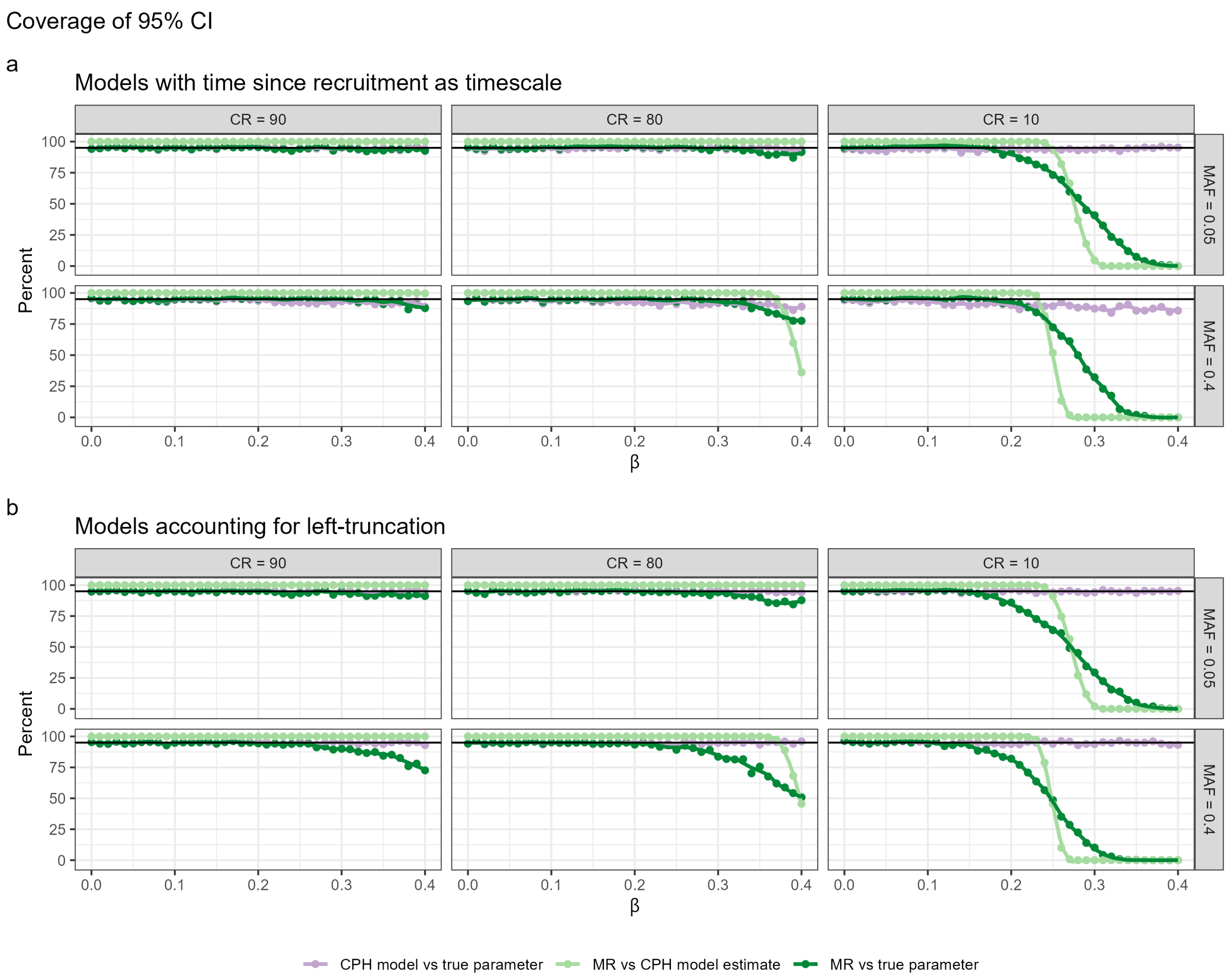

Figure 4: Coverage of 95% CI for models using time since recruitment as the timescale (a) and models accounting for left-truncation (b), evaluated across different censoring rates and MAFs. Three different scenarios are compared: β estimate using MR approach vs true β *(MR vs β)*, β estimate using MR approach vs CPH estimate *(MR vs CPH model estimate)* and CPH model estimate vs true β *(CPH model vs β)*

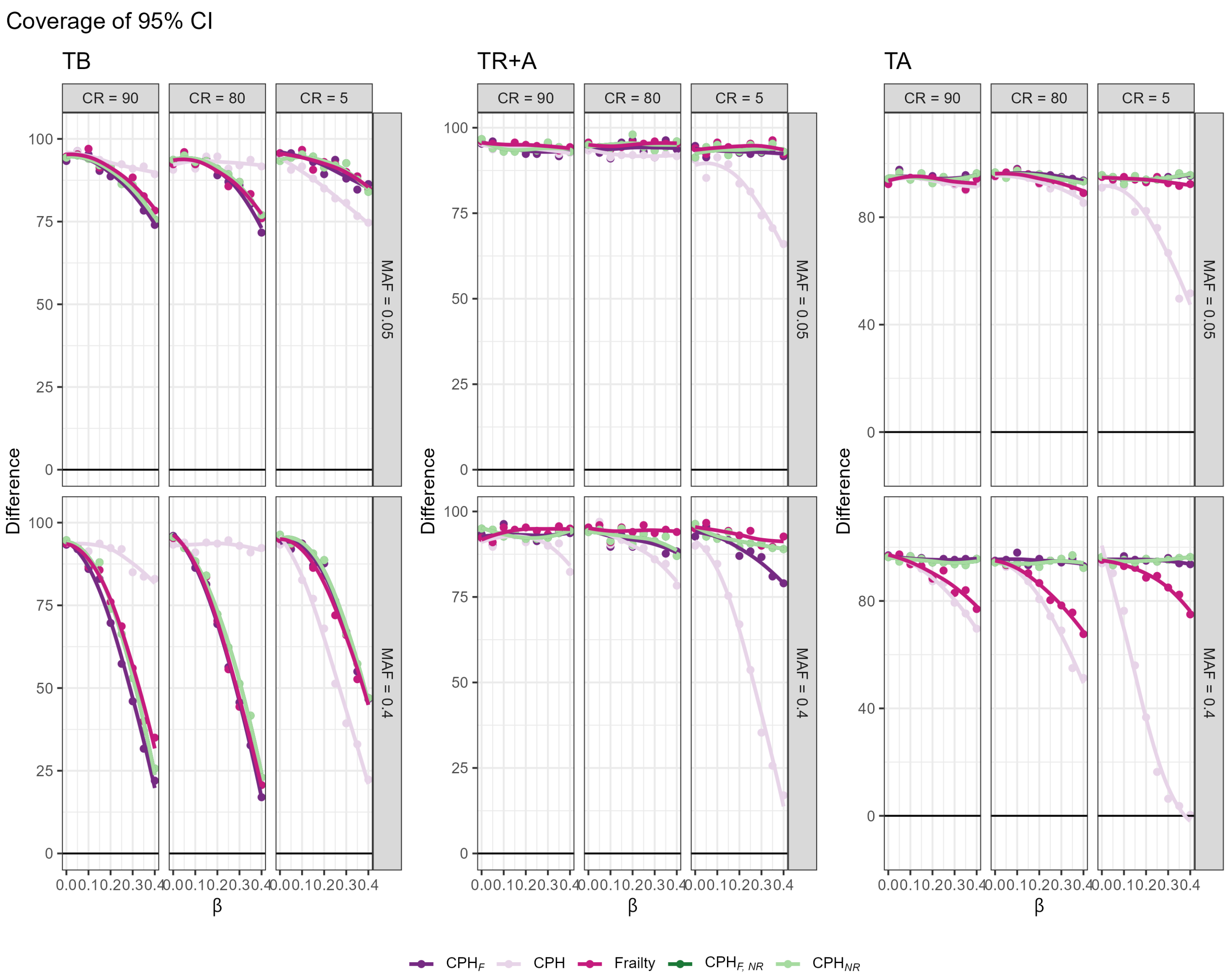

Figure 5: Coverage of 95% CI of various models across three timescales (TB, TR+A, TA), different censoring rates (CR), and MAFs. The models include full covariate models with all subjects (CPH_F) and only independent subjects ($CPH_{F,NR}$), models omitting the frailty term for all subjects (CPH) and only independent subjects (CPH_{NR}), and a CPH frailty model for all subjects (*Frailty*).

Table 4: Comparison of hazard ratios for survival-associated SNPs using CPH with different timescales and related vs unrelated participants in the Estonian Biobank data. TB, TR+A and TA correspond to CPH using time since birth as timescale, time since recruitment + age adjustment as timescale and age as timescale with all EstBB participants. The estimates for Timmers et al. can be found here: https://doi.org/10.7554/eLife.39856.015.

SNP	Model	TB (related)	TB (unrelated)	TR+A (related)	TR+A (unrelated)	TA (related)	TA (unrelated)	Timmers
ATXN2/BRAP	HR	1.00	0.99	1.01	1.00	1.01	1.00	
	CI	(0.96, 1.04)	(0.95, 1.04)	(0.97, 1.05)	(0.96, 1.05)	(0.97, 1.05)	(0.95, 1.04)	
CHRNA3/5	HR	1.03	1.02	1.02	1.01	1.02	1.01	1.04
	CI	(0.93, 1.01)	(0.94, 1.03)	(0.94, 1.02)	(0.94, 1.03)	(0.94, 1.02)	(0.94, 1.03)	(1.02, 1.05)
FURIN/FES	HR	0.97	0.98	0.98	0.99	0.98	0.99	0.97
	CI	(0.98, 1.06)	(0.96, 1.05)	(0.97, 1.05)	(0.95, 1.04)	(0.97, 1.05)	(0.95, 1.04)	(0.96, 0.98)
HP	HR	0.98	0.99	0.97	0.98	0.97	0.98	0.97
	CI	(0.94, 1.03)	(0.94, 1.04)	(0.93, 1.02)	(0.93, 1.03)	(0.93, 1.02)	(0.93, 1.03)	(0.96, 0.98)
LDLR	HR	0.98	0.98	1.00	0.99	0.99	0.98	0.97
	CI	(0.92, 1.05)	(0.91, 1.05)	(0.94, 1.07)	(0.92, 1.07)	(0.94, 1.06)	(0.91, 1.06)	(0.95, 0.98)
APOE	HR	1.12	1.09	1.09	1.06	1.10	1.07	1.08
	CI	(1.06, 1.18)	(1.02, 1.16)	(1.03, 1.15)	(0.99, 1.12)	(1.04, 1.16)	(1.00, 1.13)	(1.05, 1.11)
MAGI3	HR	0.98	0.97	0.98	0.97	0.98	0.97	0.97
	CI	(0.94, 1.03)	(0.92, 1.03)	(0.93, 1.03)	(0.91, 1.02)	(0.93, 1.03)	(0.92, 1.03)	(0.95, 0.98)
KCNK3	HR	1.00	1.00	1.00	1.01	1.00	1.00	1.03
	CI	(0.96, 1.04)	(0.95, 1.05)	(0.96, 1.04)	(0.96, 1.05)	(0.96, 1.04)	(0.96, 1.05)	(1.01, 1.04)
HTT	HR	0.98	0.99	0.99	1.00	0.98	1.00	0.98
	CI	(0.94, 1.02)	(0.94, 1.03)	(0.95, 1.02)	(0.95, 1.04)	(0.95, 1.02)	(0.95, 1.04)	(0.96, 0.99)
HLA-DQA1	HR	0.93	0.94	0.94	0.94	0.93	0.94	
	CI	(0.88, 0.99)	(0.88, 1.01)	(0.88, 0.99)	(0.88, 1.01)	(0.88, 0.99)	(0.88, 1.01)	
LPA	HR	1.02	1.01	1.02	1.01	1.02	1.01	1.09
	CI	(0.94, 1.11)	(0.92, 1.11)	(0.94, 1.10)	(0.92, 1.10)	(0.94, 1.10)	(0.92, 1.11)	(1.06, 1.13)

Note: the 11 tested SNPs were chosen based on Timmers et al Table 1 (https://doi.org/10.7554/eLife.39856.003). However, as these results do not contain hazard ratios, the corresponding results were extracted from Table 2 Source data 2 (https://doi.org/10.7554/eLife.39856.015).
